# Accumulation of Splice Variants and Transcripts in Response to PI3K Inhibition in T Cells

**DOI:** 10.1371/journal.pone.0050695

**Published:** 2013-02-01

**Authors:** Alice Riedel, Boitumelo Mofolo, Elita Avota, Sibylle Schneider-Schaulies, Ayton Meintjes, Nicola Mulder, Susanne Kneitz

**Affiliations:** 1 Institute for Virology and Immunobiology, University of Wuerzburg, Versbacher, Wuerzburg, Germany; 2 Department of Physiological Chemistry I, Biocenter, University of Wuerzburg, Wuerzburg, Germany; 3 Computational Biology Group, Department of Clinical Laboratory Sciences, Institute of Infectious Disease and Molecular Medicine, University of Cape Town, Faculty of Health Sciences, Cape Town, South Africa; National Cancer Institute, National Institutes of Health, United States of America

## Abstract

**Background:**

Measles virus (MV) causes T cell suppression by interference with phosphatidylinositol-3-kinase (PI3K) activation. We previously found that this interference affected the activity of splice regulatory proteins and a T cell inhibitory protein isoform was produced from an alternatively spliced pre-mRNA.

**Hypothesis:**

Differentially regulated and alternatively splice variant transcripts accumulating in response to PI3K abrogation in T cells potentially encode proteins involved in T cell silencing.

**Methods:**

To test this hypothesis at the cellular level, we performed a Human Exon 1.0 ST Array on RNAs isolated from T cells stimulated only or stimulated after PI3K inhibition. We developed a simple algorithm based on a splicing index to detect genes that undergo alternative splicing (AS) or are differentially regulated (RG) upon T cell suppression.

**Results:**

Applying our algorithm to the data, 9% of the genes were assigned as AS, while only 3% were attributed to RG. Though there are overlaps, AS and RG genes differed with regard to functional regulation, and were found to be enriched in different functional groups. AS genes targeted extracellular matrix (ECM)-receptor interaction and focal adhesion pathways, while RG genes were mainly enriched in cytokine-receptor interaction and Jak-STAT. When combined, AS/RG dependent alterations targeted pathways essential for T cell receptor signaling, cytoskeletal dynamics and cell cycle entry.

**Conclusions:**

PI3K abrogation interferes with key T cell activation processes through both differential expression and alternative splicing, which together actively contribute to T cell suppression.

## Introduction

Suppression of T cell activation and function ranks amongst the most powerful strategies of viruses to modulate host responses. In particular, viruses establishing persistent infections in their hosts exploit numerous strategies to prevent activation of virus-specific helper or effector T cells by interfering with processing and/or presentation of viral peptides on MHCI or II molecules, and thus evade immune recognition (for a recent review see [1]–[4]). Much less frequently, viruses may also cause a generalized, non-specific suppression of T cell activation, and in humans, both HIV and measles virus (MV) are paradigms of this scenario. In HIV infection, immunosuppression is maintained and progressive, while that induced by MV is transient, yet also almost exclusively accounts for the continuously high rates of morbidity and mortality associated with the acute disease [5]–[7]. MV immunosuppression is typically associated with an expansion block of polyclonally or antigen-specific stimulated peripheral blood T cells, the vast majority of which are uninfected, indicating that they were actively silenced [7]. Soluble mediators accounting for MV T cell silencing have not been detectable, yet both in vitro and animal experimentation suggests that MV proteins can act as effectors interfering with T cell activation [8]–[10].

The highly efficient, generalized inhibition of T cells by MV suggests that pathways centrally involved in relaying T cell receptor (TCR) signaling to promote cell cycle progression would be targeted independently of direct infection of T cells. Indeed, the MV glycoprotein complex interacts with an unknown receptor to abrogate S phase entry of primary T cells in vitro and in vivo [11], [12]. On a molecular level, TCR driven activation of the phosphatidylinositol-3(PI3)/Akt kinase pathway, which is key to T cell activation and expansion, was identified as a prime target of MV mediated inhibition, and consequently, overexpression of a constitutively active Akt protein alleviated MV T cell paralysis to a major extent [13]. Lack of Cbl-b degradation and activation of sphingomyelinases are apparently upstream of MV surface interaction mediated PI3K interference [14], [15], yet specific molecular targets supporting this particular mode of T cell silencing remained largely undefined. They may include regulation of activity and/or subcellular redistribution of known PI3K downstream targets such as the Vav, GSK-3b, and FOXO1, the importance of which in T cell S phase entry is established [16], [17]. In line with earlier observations [18]–[20], we confirmed that certain splicing accessory factors are subject to PI3K interference, induced either by MV or on pharmacologic PI3K inhibition by LY294002 or wortmannin in T cells. Importantly, PI3K interference by either means gave rise to production of a SHIP145 5-phosphatidylinositol-phosphatase isoform, SIP110, which was produced from intron-retaining *SHIP145* mRNA, in T cells. When ectopically expressed from cloned cDNA, SIP110 interfered with TCR-driven expansion and thus acted as a T cell silencer [21]. This finding suggested that PI3K targets altered at the level of alternative splicing on inhibition of the enzyme might have this activity and SIP110 identified by chance on MV interruption of this pathway might represent an example of others yet to be identified.

Differentially regulating the output of up to 94% of human gene transcripts, alternative splicing (AS) generates diversity in the human proteome by inclusion or exclusion of exonic/intronic sequences and thereby mRNA stability and the composition of functionally organized protein domains (for review see [22], [23]). While AS has been recognized in T cell differentiation and activation, little is known of its functional importance [24]–[27]. The number of genome-wide analyses of AS in these cells is limited, and alterations thereof in response to immunosuppressive conditions have not yet been reported. Because of its key role in controlling cell cycle entry and cell survival, transcripts accumulating in response to PI3K inhibition in activated T cells may serve as T cell quiescence biomarkers or have the potential to act as T cell silencers. Because both transcription and splice accessory factors are PI3K effectors [16], [18]–[20], [28], [29], alterations in the T cell transcriptome on inhibition of the enzyme would be expected to occur at the level of overall gene regulation, yet also alternative splicing (AS), as already evidenced for *SHIP145*. To investigate early alternative splicing (AS) and differential gene expression in response to PI3K interference in T cells at a general level, we performed GeneChip Exon array analysis on RNAs isolated from human T cells pre-exposed to LY294002, or not, prior to a 24 h phorbolester/ionomycin exposure. Based on existing algorithms for the detection of alternative splicing events we developed a simple method to identify exons that are enriched under treatment. Transcripts detected specifically in PI3K inhibited cells were assigned to categories defining either differentially regulated (RG) (619 candidates) and alternatively spliced (AS) species (2192 candidates). These gene lists were analyzed for functional annotation and representation in molecular networks and pathways, and selected transcripts from these lists were validated by RT-PCR and qPCR.

## Methods

### Lymphocyte isolation, stimulation, RNA extraction and quality control

Human peripheral blood mononuclear cells obtained from healthy donors served as the source for T cells. These were enriched on nylon wool columns (purity of CD3+ cells: on average 85%). Individual samples maintained in RPMI1640/10% FCS were halved: one half was stimulated for 24 h with 40 ng/ml PMA (Calbiochem) and 0.5 µM ionomycin (Sigma) (stimulated only, S), the other half was stimulated after a 2 h exposure to 50 µM LY200492 (Calbiochem) For the inhibited/stimulated samples, the abbreviation I is used to avoid confusion with SI (splicing index) and IS (immunological synapse). Total RNA was extracted using an RNeasy Mini Kit (QIAGEN). The integrity of the RNAs was controlled by gel analysis followed by determining RNA integrity numbers (RIN) using a Bioanalyzer (Agilent, Santa Clara, CA) for those RNAs that were utilized for the Exon array (RIN 8.6 to 9.7 were used).

The use of the human material was approved by the ethics committee of the University of Wuerzburg. Because only cells are used and are without identifiers, this work is exempt from institutional review board review.

### GeneChip® Human Exon 1.0 ST array profiling

cDNA and cRNA synthesis of 100 ng of total RNA of both S and I cells was processed according to the manufacturer's (Affymetrix, Santa Clara, CA) instructions and hybridized on GeneChip Human Exon 1.0 ST Arrays (Affymetrix). Hybridization, washing and scanning were carried out according to manufacturer's (Affymetrix) instructions. Data are deposited in GEO, with accession number GSE38255.

### Selection of candidate genes

Probe set signal intensities were summarized using the Expression Console software (Affymetrix). The probe set summarization algorithm PLIER was used for the exon level and iterPLIER for gene level analysis. In both cases only probe sets annotated as “core” were included. For all further analysis the statistical language R, including different packages from the Bioconductor project (www.bioconductor.org), was used. For quality control, scale factor, provided by the Affymetrix system, degradation plot and correspondence analysis were evaluated. Exon level data and gene level data were normalized by quantile-quantile normalization [30].

#### Prefiltering

Low intensity gene/exons, defined as having an average signal intensity of both groups below the 10%-quantile of the average total intensities of all samples, were excluded to correct for “unexpressed” genes and poorly performing genes/exons. Genes/exons exhibiting strong individual variations (within group variance >1) and thus having poor reproducibility were excluded. For the detection of splice variants, only exons having a corresponding gene value were included. Next, the average log2(ratios) between I and S were calculated at the probeset level as well the gene level and a splicing index (SI) was calculated for each probeset as the difference between probeset ratio and the corresponding gene ratio [31].

Alternatively spliced (AS) candidate genes: Assuming that alternative splicing correlates with the extent of SI divergence, the range of all SIs of a corresponding gene was calculated and a range (max SI – min SI) cutoff of 1 was applied to each gene. In previous experiments we observed a slightly higher variation of signal intensities in Affymetrix exon arrays compared to 3′IVT arrays. This may be because the selection of optimal probesets is more difficult due to closer spacing [32]. To avoid false positive AS genes due to possible outliers, we applied a sliding window approach with a window size of 3 to emphasize longer stretches of equally regulated probesets and to down-weigh single peaks. This sliding window was applied to the selected genes comprising more than 6 probesets (2× window size), followed by a subsequent second filtering using a range-cutoff of 0.75. To test for dependencies between changes in transcription rate and exon inclusion as described [32] in several microarray samples, we plotted SI against logFC, but detected no visible bias (data not shown).

#### Regulated candidate genes (RG)

**Figure 1:**
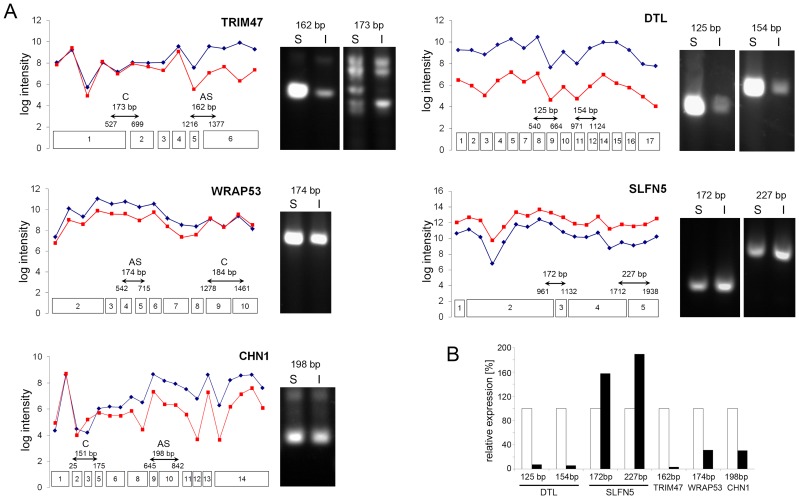
Selected AS (*TRIM47*, *WRAP53*, *CHN1*; left) and RG (*DTL*, *SLFN5*; right) gene views showing probe set intensity plots (graphs) for each stimulated only (S, blue) or stimulated/inhibited (I, red) samples. Exons assigned with probe sets, location of primers within constant and AS regions and expected fragment lengths on RT-PCR analyses (which are exemplified each right to the corresponding graphs; one out of at least three independent experiments is shown) are indicated below the transcript profiles (A). Samples prepared as in A. were used for qPCR analyses with amplification levels for S (white bars) and I (black bars) being indicated. Data shown were obtained using a selected pair of RNAs (S and I), assays were performed in triplicate (B).

All expressed genes were used, regardless of a corresponding transcript ID within the exon list. In a first approach, for the calculation of the log fold change and p-value we used the Limma (Linear Models for Microarray Analysis) package [33] to compute a modified t-test, based on the normalized core gene level data. A gene was defined as differentially regulated, if the fold change was above 1.5 and p-value<0.01. However, taking the average intensity of all exons was susceptible to false positives in the case of genes being strongly spliced. For example, TRIM47 showed a gene log fold change of −1.67 and an adjusted p-value of 0.0004. However, only exons 5 and 6 display a significantly lower expression in the inhibited samples (Figure 1). Therefore, in a second approach, we calculated the modified t-test on the normalized core exon level data. An exon was defined as differentially expressed, if the fold change was above 1.35 with an overall fold change for a gene >1.35. We decided to lower the required fold change compared to gene wise calculation, because of the higher number of observations. In addition and in accordance with the definition of a gene being spliced, at least 90% of all exons were required to be differentially expressed. Finally, spliced genes were excluded from the list.

### Functional analysis of gene lists

Functional analysis was performed using web-based tools, including DAVID (http://david.abcc.ncifcrf.gov/tools.jsp), for functional clustering, the Gene Set Analysis Toolkit V2 (http://bioinfo.vanderbilt.edu/webgestalt), for GO term enrichment analysis, and KEGG Mapper (http://www.genome.jp/kegg/tool/color_pathway.html) for pathway mapping using the default settings. Additional functional clusters and text mining for gene interactions were generated through the use of IPA (Ingenuity Systems, www.ingenuity.com).

### Identification of T cell suppression genes from public databases

Previously predicted T cell suppression genes were extracted from public databases by extracting microarray datasets from GEO (http://www.ncbi.nlm.nih.gov/geo/) and ArrayExpress (www.ebi.ac.uk/arrayexpress), transcript data from dbEST (www.ncbi.nlm.nih.gov/dbEST), and alternative splicing data and information from Ensembl (www.ensembl.org), Genecards (www.genecards.org) and Entrez (http://www.ncbi.nlm.nih.gov/Entrez). Gene lists were generated to identify genes overexpressed during T cell suppression, as well as those showing alternative transcripts under normal versus T cell suppression conditions (Table S1). These lists were compared to those obtained from the exon array data.

### Class validation by semiquantitative RT-PCR and qPCR

For RT-PCR, cDNA was synthesized from 2 µg total RNA (S or I) by oligo-d(T) primed reverse transcription using the First Strand cDNA Synthesis Kit (Fermentas). Illustra PuReTaq Ready-To-Go PCR beads (0.5 ml tubes, GE healthcare) were used to amplify *DTL*, *SLFN5*, *TRIM47*, *WRAP53* and *CHN1* transcripts (denaturing at 94°C for 30 s, annealing at 58°C for 1 min and elongation at 72°C for 2 min, 25–35 cycles). For the RG *DTL* and *SLFN5*, two primer pairs were used spanning two arbitrarily chosen exon boundaries and for the AS *TRIM47*, *WRAP53* and *CHN1*, one primer pair spanning exon boundaries within the predicted AS region and one primer pair within the constant region were used (see Table S2 for primer sequences, locations and expected fragment lengths). *β-actin* served as the control for the RT-PCRs (FW 514 bp 5′ GCACTCTTCCACCTTCCTTCC, REV 514 bp 5′ TCACCTTCACCGTTCCAGTTTT). Primers used in *CD44* exon specific PCRs were as follows: v3 (variable exon 3, also equating to exon 8 (out of 20)),: 5′- CAGGCTGGGAGCCAAATGAAGA-3′ and 5′-TGTTCA-CCAAATGCACCATTTCC-3′ (constant exon 19 (c19) specific); c5, 5′-TTTACACCTTTTCTACTGTACACC-3′ and exon c19 specific reverse primer. The PCR products were separated on a 2.5% agarose gel containing GelRed (1∶30000, Genaxxon) for visualization. When indicated, AIDA software was used to quantify signal intensities to allow for semi-quantitative analyses by normalizing transcript specific signals to those of actin, and subsequently, to compare S and I samples.

For qPCR the ColorFlash SYBR® Green qPCR Kit (F-416, Finnzymes) was used to prepare mastermixes, each containing 1× SYBR Green Mastermix, each 500 nM forward and reverse primer (same primers as for RT-PCR), 12.5 nM Fluorescein Calibration Dye (BioRad) and DEPC-H_2_O up to 19 µl (final volume). 1 µl cDNA (S or I) was applied in triplicate to wells of a 96-well plate as was a no template control (H_2_O). Following addition of 19 µl mastermix to each PCR template, the following iCycleriQ® experimental protocol was used: denaturation (95°C for 7 min), amplification and quantification repeated 45 times (95°C for 10 s, 60°C for 30 s), melting curve (52 ¨C 95°C with a heating increase of 0.5°C every 10 s) and a final cooling step to 15°C. For a control and quantification, the Quantitect Primers UBC (ubiquitin C) and YWHAZ (tyrosine-3-monooxygenase/tryptophan5-monooxygenase activation protein, zeta polypeptide) were used (Qiagen).

**Figure 2:**
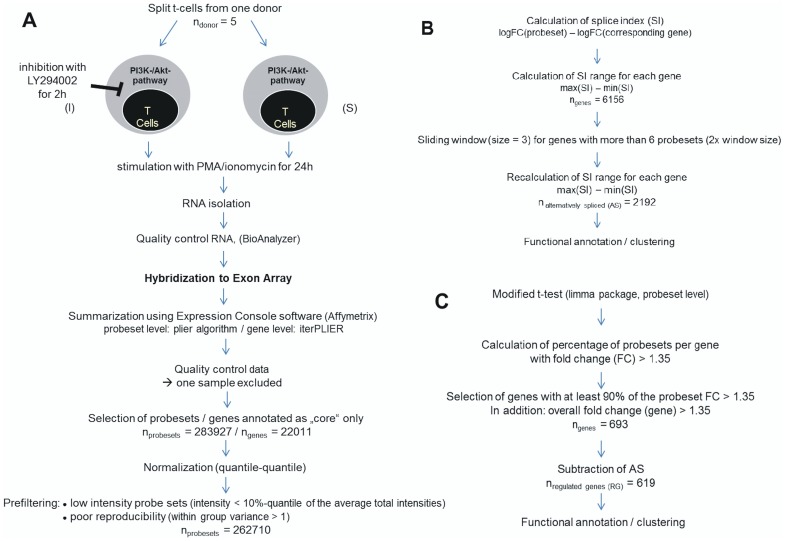
Overview of cell treatment, hybridization and prefiltering of the resulting data sets (A), selection of alternatively spliced genes (B) and selection of differentially expressed genes (C).

The ΔΔCT-value was determined by the iCycler program and used for comparative quantification by the ΔΔCT- method. This method is suitable for a quick estimation of the relative expression ratio between two groups (S or I), but assumes optimal and identical amplification efficiencies of target and reference genes. The use of two housekeeping genes, which allowed for exact quantification, required normalization with the geometric mean. An overview of cell treatment, hybridization and data analysis steps is given in Figure 2.

## Results

### Identification and categorization of RG and AS gene products as a function of PI3K inhibition in activated T cells

Our work piloting this study revealed that MV interaction with an as yet unknown surface receptor abrogates activation of the PI3K by TCR signaling, and as a consequence of this, alters the activity of splice regulatory factors and production of a fully spliced mRNA for SHIP145 in T cells [21]. To assess alterations of genome-wide early transcription upon suppression of PI3K signaling in activated T cells, we decided to use a robust stimulation protocol (phorbolester/ionomycin, P/I) which fully reproduces TCR-mediated activation performed by CD3/CD28 crossligation in the pilot study. We also decided to replace MV by a commercial inhibitor (LY294002, targeting the ATP-binding site of the PI3K) for abrogating PI3K activation (added 2 h earlier) to avoid virus preparation related variances (such as purity and concentration), but also to cope with potential differences in expression levels and membrane density of the unknown receptor used by MV for PI3K interference. To record efficient P/I activation of T cells, *CD44* exon V3 exclusion known to occur on T cell stimulation was followed at various time intervals by RT-PCR (Figure S1). Thus, for array analysis, T cells enriched from PBMCs of 7 independent healthy donors were each halved with only one aliquot being exposed to LY prior to P/I stimulation to obtain stimulated (S) and stimulated/inhibited (I) samples. RNAs were isolated from paired samples after 24 h, and, following quality control by RIN level determination, 2 paired samples were discarded, and the five remaining pairs (S and I per donor) were used for analysis with Affymetrix Human Exon 1.0 ST arrays, which contain multiple probe sets interrogating all currently known exon sequences in the human genome.

### Extraction of candidate genes and class assignment

Using core probe sets only and discarding exons as well as transcripts unexpressed in both groups, 283,927 probe sets corresponding to transcript cluster IDs remained for filtering after matching probe set and transcript IDs. Filtering for low expressed probe sets also removed effects such as non-responsive probe sets that might result in an increased number of false positive, as described in [34]. One sample was identified as an outlier by quality control plots, thus, the pair was not recruited into the analysis. To select alternatively spliced genes, in a first step, a splice index (SI) [31] for each probe set was calculated as the log ratio (logFC) of the probe set intensities and gene intensities. Based on the idea that the likelihood of alternative splicing of a gene correlates with variation levels of the SIs of its probe sets, the SI range for each gene was determined. Applying the filters described in the methods section, 6156 transcripts were categorized as being AS. To further increase stringency and to emphasize consecutive probe sets being regulated in the same direction, a sliding window was applied to each transcript represented by more probe sets than twice the window size (window size = 3). After a second filter step 2192 genes were finally assigned as AS (Table S3). Calculating a modified t-test using the Bioconductor package Limma for the selection of differential exon signals and selecting regulated genes (RG) resulted in a total of 619 genes, 280 of which were up-regulated and 373 were down-regulated (Table S4).

### Validation of class assignment

Based on the distribution of log signal intensities and splice indices of the probesets, 9 candidate genes were chosen from the RG and 7 from the AS class list for RT-PCR analyses (Table 1). To validate assignment to the RG class, primers were randomly chosen for two independent regions (exemplified in Figure 1A, right graphs, denticeless homolog (*DTL*) [35] and Schlafen5 (*SLFN5*) [36]), while for the AS class, primers were designed within the AS and the constant region (for examples see Figure 1A, left graphs, tripartite motif containing 47 (*TRIM47*) [37], WD repeat domain 79 (*WRAP53*) [38], and chimerin 1 (*CHN1*) [39]). Two-step RT-PCRs were performed using cDNAs prepared from paired RNA samples used to probe the Exon arrays, and additional pairs prepared according to the same stimulation/inhibition protocol. RT-PCR based validation always confirmed assignment of RGs to this class. Thus, when normalized to the respective actin-specific amplificate, both amplificates of the RG analyzed were down- or up-regulated upon LY294002 treatment as exemplified for *DTL* (about 45% down) and *SLFN5* (about 130% up) (Figure 1A, right panels). These semi-quantitative analyses were quantitatively confirmed by qPCR (Figure 1B). Similarly, validation of predicted and visually inspected AS transcripts both by RT-PCR (Figure 1A, left panels) and qPCR (Figure 1B) confirmed differences in the accumulation levels of transcript amplified from within the AS region (49% for TRIM47, 83% for WRAP53 and 85,2% for CHN1). For regions predicted not to be subjected to alternative splicing, results were quite unambiguous because in most cases, differences with regard to accumulation levels were seen (not shown). However, because relative accumulation levels between the AS and the constant region were unrelated (in contrast to RG transcripts), assignment of the transcripts to the AS class appeared valid.

**Table 1:** RG and AS class transcripts RT-PCR amplified for validation of class assignment performed on a representative number of independent RNAs for RGs.

class	gene symbol	alternative name	gene function	class confirmed
**RG**				
	SLFN5	Schlafen 5	unknown	yes
	DTL	denticeless	E3 protein ligase homologue	yes
	PFKM	PFK1	phosphofructokinase	yes
	DOK2	p56DOK2	RasGAP adapter	yes
	ARHGEF6	COOL2	Rac/Cdc42 guanosine exchange factor	yes
	RAD51AP1	PIR51	RAD associated protein 1	yes
	HELLS	LSH	lympoid specific helicase	yes
	DNMBP	TUBA	dynamin binding protein	yes
	SESN3	Sestrin 3	regulator of TORC activity	yes
**AS**				
	TRIM47	Tripartite motif-containing protein 47	unknown	V: 10/10
				C: 9/10
	WRAP53	WD repeat containing, antisense to p53	p53 regulator, telomerase complex	V: 5/7
				C: 1/7
	CHN1	Chimerin 1	RhoGTPase activator	V: 5/5
				C: 2/5
	TPRG	p63 regulated gene 1 protein	unknown	V: 4/6
				C: 2/6
	STAP2	Substrate of breast cancer kinase	adaptor	V: 7/7
				C: 3/7
	IL4I1	Fig1, IL-4 induced protein 1	L-phenyl-oxidase, inhibition of T cell proliferation	V: 4/6
				C: 2/6
	FBX06	F box protein 6	Subunit of the SKP1/cullin-F-box complex, ubiquitination	V: 5/8
				C: 5/8

The frequencies of AS transcript analyses per variable (v) or constant (c) regions are indicated, and those matching AS criteria (differing in the AS region and regulated differently than the AS region within the C region) are indicated. Bold gene symbols mark genes for which, based on Gene annotation, as yet no alternatively spliced transcripts were described.

### Functional analysis

**Figure 3:**
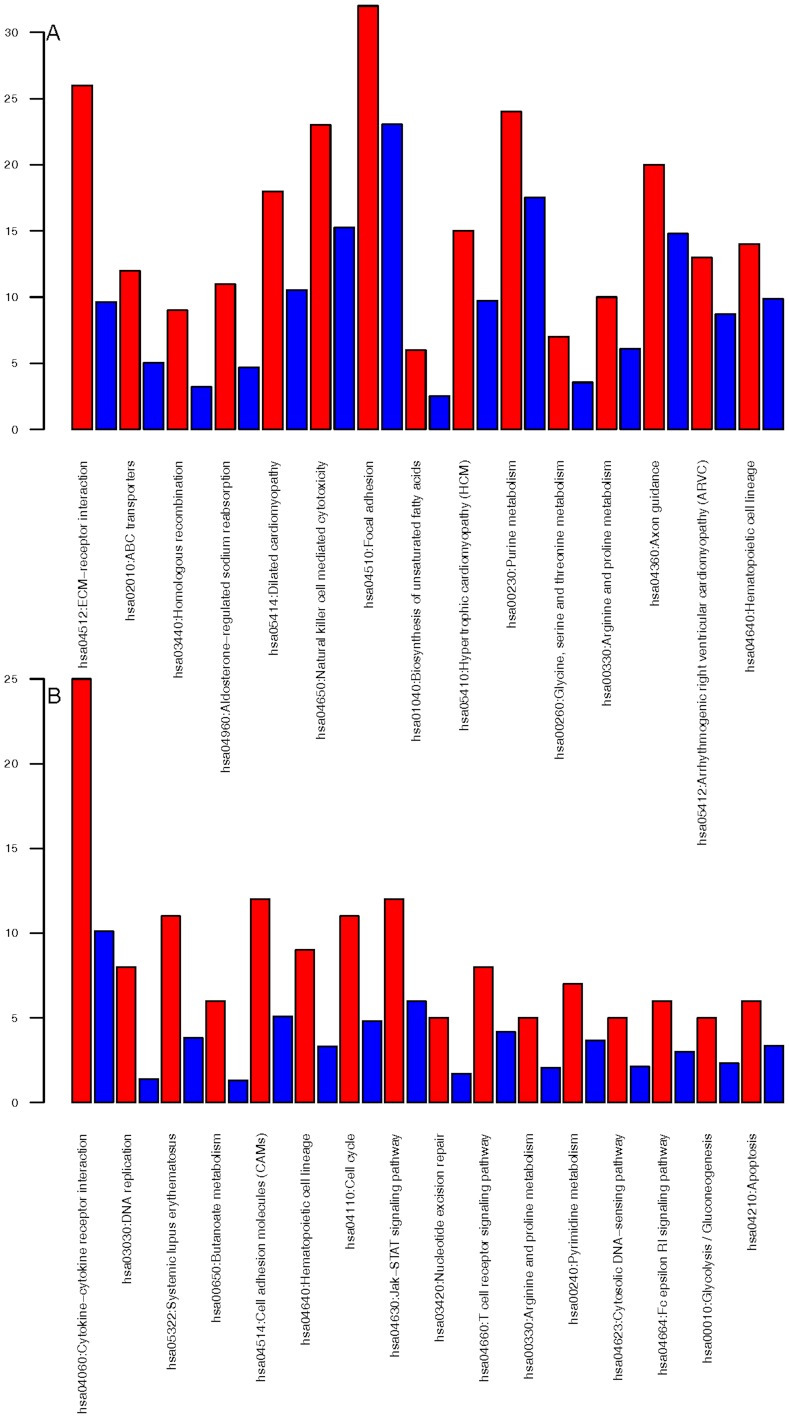
Affected pathways based on SI genes (A) or genes commonly up-regulated or down-regulated (B). Red bars show the number of observed genes in the dataset, blue bars show the statistically expected number of genes, given the result to be random.

For pathway assignment using DAVID, pathways showing a p-value<0.01 and containing a minimum number of genes for the corresponding term of 2, were considered to be significantly enriched. Pathway analysis revealed that AS genes appeared to be preferentially associated with extra cellular matrix (ECM)-receptor interaction and focal adhesion, purine metabolism, and natural killer cell mediated cytotoxicity, while RGs were major targets in cytokine-receptor interaction, the Jak-STAT pathway and DNA replication (Figure 3). Combining both AS and RG genes, functions involved in signaling, cell cycle, and DNA replication, recombination and repair were mainly affected. In concordance with functional clustering using DAVID, text mining resulted in networks including cell cycle with the highest scores, which is in line with the established importance of PI3K signaling in cell cycle progression in T cells [40] [41] [42].

The Gene Ontology (GO) analysis identified RG genes as being enriched in immune response and DNA replication, whereas AS genes were preferentially related to cell migration and motility processes. Cell cycle regulation was enriched in both classes of genes in the GO analysis. At the level of subcellular distribution, AS tended to be enriched at the plasma membrane (encoding receptors and ECM interaction), while RGs, irrespective of whether they were up- or down-regulated, were more commonly found in association with intracellular organelles and related biochemical processes (Figure S2, S3 and S4). When analyzed separately by GO analysis, down-regulated RGs were significantly overrepresented in pathways regulating immune response, cytokine activity, DNA replication and response to DNA damage, while up-regulated RGs were overrepresented in DNA assembly and packaging, and receptor and protein kinase activity (*MAP3K3, TNK, CLK1, TESK2, SGK3, CDKL5, NUAK2*). KEGG pathways over-represented in the up-regulated set include SNARE interactions in vesicular transport (hsa04130) and the p53 signaling pathway (hsa04115), while those in the down-regulated set include cytokine–cytokine receptor interaction (hsa04060), DNA replication (hsa03030), and the Jak–STAT signaling pathway (hsa04630). On specific interrogation of the combined AS and RG lists, 15 members of TNF/TNF-R family were found targeted on PI3K ablation (Table 2), and interestingly, only three of them (*FAIM3*, *TNFSF8* and *TNFRSF10D*) were down-regulated while all others were AS.

**Table 2:** TNFSF/TNFR-SF related genes affected on PI3K disruption in T cells.

Gene symbol	alternative name	list	variant known
TNFSF8	CD30L,	RG	yes
TNFSF11	RANKL, TRANCE, CD254	AS	no
TNFSF12	APO3L, TWEAK,	AS	yes
TNFSF13	APRIL, CD256	AS	yes
TNFSF15	VEGI, TL1	AS	yes
TNFSF18	GITRL	AS	no
C1QTNF5	LORD, C1Q and TNF related 5	AS	no
C1QTNF6	CTRP6	AS	yes
C1QTNF7	CTRP7	AS	yes
TNFRSF8	CD30, Ki-1	AS	yes
TNFRSF10D	TRAIL-R4, CD264	RG	no
TNFRSF21	DR6, CD358	AS	no
TNFRSF25	DR3, APO-3, TRAMP	AS	yes
FAIM3	FcR for IgM, TOSO	RG	yes
TNFAIP8L2	TNF-a-induced protein 8-like2	AS	no

Overlaps between the gene lists and previously published data on genes responsive to PI3K activity alterations resulting from the text search tool within IPA include for the AS list: *CYBB*, *MAPK3*, *NOS3* and *TERT* and for the RG genes: *CDKN1A* (p21^WAF^), which, in line with its CDK inhibitory activity, is strongly up-regulated. Because PI3K activity abrogates that of FOXO1, FOXO1 targets such as *SESN3*, *CDKN1A*, *SAT1*, and *VCL* were found up-regulated [43] [44],[45] while other typical FOXO1 targets, such as *FASL* and *P27^KIP^* were not detectably affected. Corroborating the role of PI3K in cell cycle progression, bcl-family members (*BCL-11B, BCL-2, BCL-3, and BCL-9L*) and transcripts coding for cyclin-dependent kinase inhibitors (p16INK4a and p18INK4b) were found altered, yet interestingly, at the level of differential exon inclusion.

### RG and AS gene representation in public data on T cell suppression

Because they are detected by the array, genes identified by the assay as a whole after filtering conditions should be expressed in the RNA samples analysed. These were lymphocytes enriched for T cells (on average 85% CD3+ cells), yet also contained contaminating B and NK cells. We thus aimed to relate findings from our analyses to genes known to specifically be associated with key T cell functions and viability. For this, we first determined the overlap of our gene lists with those containing gene functions important in chemokine signaling (hsa4062), leukocyte transendothelial migration (hsa04670) and TCR signaling (hsa04660), which yielded an overlap of 18 genes for RG and 24 genes for AS (Table S5).

**Figure 4:**
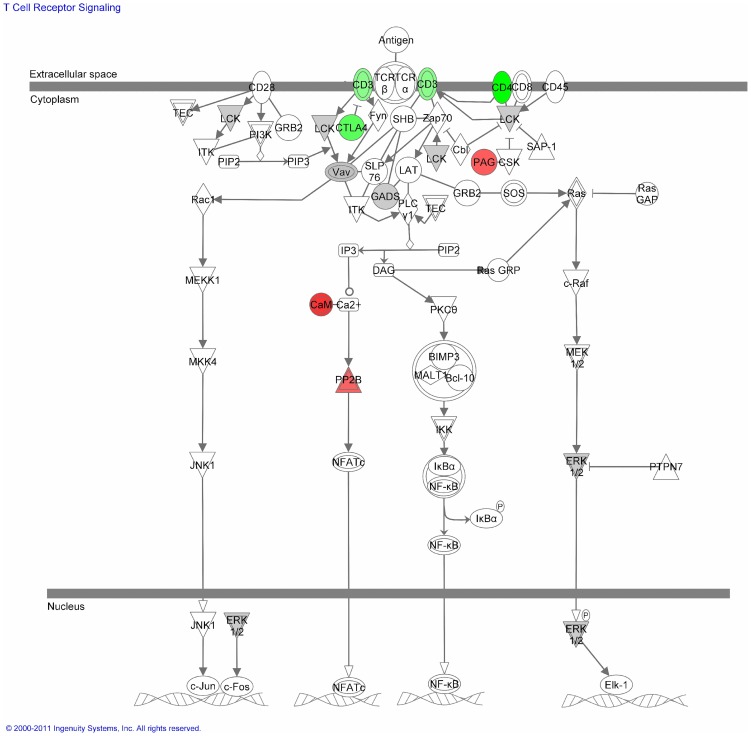
T cell receptor signaling pathway. Green indicates a down-regulated gene, red indicates an up-regulated gene. Spliced genes are marked in grey.

Of those, several are directly implicated in perception and relay of TCR signaling (Figure 4). Both *CD3G* (encoding the γ component of the CD3 TCR complex) and *CD4* were found to be down regulated, while *LCK*, an essential kinase in propagating TCR signaling, was targeted at the level of AS. Interestingly, *LCK* transcript variants initiated at alternative transcription start sites are described, yet so far, alternative splicing has not been linked to variants at the protein level. A group of affected genes relevant to TCR signaling includes the AS guanosine exchange factors (*VAV1*, *VAV3*, *TIAM1* and *TIAM2*, for which splice variants are known), which play a critical role in translating extracellular signals into cytoskeletal dynamics, protein phosphatases (*PTPRC* (CD45), *PTPN6* (SHP-1) and *PPP3CA* (calcineurin A)) and adaptors (*DOK2*, *DOK5*, Shc; all AS). In addition to TCR signaling, T cell adhesion appears to be affected at multiple levels as seen through alteration at the level of receptors (*ITGAL* (LFA-1), *ITGA4* (VLA-4), *ITGAM* (integrin alphaM), *ITGB2* (MAC-1)) and GEFs, which regulate their coupling to the cytoskeleton.

### RG and AS gene representation in public databases

Assuming that at least some RG/AS genes being regulated in response to PI3K disruption might represent gene products already linked to T cell suppression, we explored microarray, EST and alternative splicing data in public databases for comparative analysis. From these gene lists, we specifically identified genes showing different transcripts under normal versus T cell suppression conditions (Table S1). In this list *ATM*, *PRMT5* and *VCL* (vinculin) overlapped with the RG list from the exon array and *CALD1*, *LCK* and *MXI1* overlapped with our AS gene list, thus providing additional evidence for these genes having different products under different conditions. ATM was found to promote T cell survival on interaction with HTLV-I p30 (KEGG pathway hsa05166) [46]. Vinculin (*VCL*) and caldesmon 1 (*CALD1*) have established roles in actin cytoskeleton regulation and stabilization during focal adhesion, migration, invasion and proliferation, although this was not yet confirmed to apply to T cells. [47] [48]. Interestingly, the non-muscle isoform of *CALD1* is produced from an AS transcript [49]. An *MXI1* isoform generated on AS has been reported earlier to antagonize Myc functions and has thus been suggested to serve as a suppressor of solid tumors [50], and there is evidence it may act as a T cell quiescence factor too [51]. Most interestingly, neuropilin-1 (encoded by the AS *NRP1*) has been directly implicated in regulating T cell activation at the level of the immune synapse (IS) by us and others previously [52],[53].

The KEGG pathway analysis showed a higher number of AS genes than expected to be involved in cancer pathways (hsa05200). The AS and RG lists were checked against the Tumor Gene Family of Databases (http://www.tumor-gene.org/TGDB/tgdb.html) and several genes overlapped, including mitogen activated protein kinases and cyclin-dependent kinases from the AS list.

## Discussion

There has been increasing interest in studying T cell transcription and alterations of it at a whole genome level and this, in common with our study, has included the Exon ST array, and deep sequencing [54] [34], [55]. The contribution of these studies to identifying key elements in T cell activation has been extremely important, yet the approach taken previously differs from our setting in that we did not evaluate stimulation dependent parameters, but rather concentrated on genes regulated downstream of PI3K to get a handle on their impact on T cell suppression. Also applicable to these studies, much work has been done on the implementation, validation and improvement of algorithms for the detection of alternative splicing. Since most algorithms, after visual inspection of probeset intensity distribution, turned out to be unsatisfactory and revealed quite a large number of false positive genes that were either regulated or not changed at all, we decided to use a more stringent algorithm instead. A higher stringency appears to be justified, because, although there is good comparability between 3′IVT arrays and exon arrays regarding selectivity and sensitivity levels on gene level [56], around a 2.2 times higher variation in exon arrays compared to 3′IVT arrays was also described [57]. This is in line with the observation in our laboratory, where quality assessment of Affymetrix ST arrays compared to 3′IVT arrays showed that ST arrays had more outliers and lower reproducibility. This may be due to a more challenging selection of optimal probesets due to closer spacing [32]. The use of random primers, which is necessary to avoid a 3′ bias of the transcripts, may also add some variation. Further, using core probesets only, resulted in a significant reduction of the probesets per exon so that an exon was only represented by one probeset. However, like in another study [57] in our dataset more than 80% of the probe level intensities for full and extended probesets were around the background level (data not shown). Thus, even though it is possible that only one probeset/exon out of a larger number of probesets is excised, we decided to focus on, longer regions showing comparable regulation as well as distinct peaks by a sliding window, where the average value of three consecutive probesets was taken as signal intensity and single peaks in either direction were leveled. This reduced the number of AS genes to approximately one third. Using gene signal intensities derived from the Affymetrix Expression Console software for the detection of regulated but not spliced genes resulted in a high number of false positive genes that were spliced. In particular, for genes having extremely high logFC values within the spliced part, averaged signal intensities tended to show large changes. We tried to avoid this problem by using the probeset intensities and the selection of genes having only little variation of their probeset logFCs. Requiring 90% of all probesets to be differentially expressed in all samples helped to evade this problem and significantly increased the correct classification rate.

Functional analysis of the AS and RG genes showed some unique features, but also significant overlaps in the pathways and GO terms. In both cases, cell cycle appeared to be significantly overrepresented. For a particular family of proteins, the phosphatidylinositol-3,4,5-trisphosphate 5-phosphatase or SHIP family, some members were shown to be RG (INPP4B and INPP5D), while others were AS (INPPL1, INPP5B and INPP5F). Splice variants of *INPP5D* (also referred to as SHIP-1) are also known, and based on inclusion of intron-derived sequences, us and others have suggested an alternatively spliced pre-mRNA as a source for translation of SIP110 [58] [21]. As only revealed for the murine *Ship1* thus far, the intron preceding exon 6 also harbors a transcriptional start site [59], [60], and therefore SIP110 may also be produced from a separate transcript which would not appear as AS in our human assay. PI3K dependent regulation of SHIP family members at different levels suggests that a combination of differential expression and alternative splicing are used to regulate T cell suppression or modulate pathways related to this process as reported earlier [55]. Targeting of lipid metabolism which plays a key role in TCR signaling may serve as an example as revealed in our study. In addition to the INPP genes referred to above, important components in sphingolipid turnover such as *CHPT1*, *GALC* and *SPHK1* or *ACER3* were also subject to AS or RG, respectively. Interestingly, shRNA mediated ablation of ACER3, which hydrolyzes unsaturated long chain ceramides and was classified as down-regulated, was previously linked to inhibition of proliferation of non-lymphoid cells [61].

In general, genes appearing as RG on PI3K disruption would be expected to include those whose expression is regulated through PI3/AktK activation. This pathway takes part in NF-kB and STAT activation, yet also in release of transcriptional repressors that are inactivated on PI3K/Akt activation, such as FOXOs [16]. Though nuclear exclusion and therefore functional inhibition of FOXO1 is strongly dependent on PI3K driven Akt activation, only a very limited number of previously identified FOXO1 targets appeared to be affected in our analysis. Though p21INK4a and sestrin 3 proved to be regulated, typical targets such as *FASL*, *CDKN1B* (p27Kip) or *TNFSF10* (TRAIL) [45],[44] were not and this may be due to the fact that they did not reach the statistical threshold in our analysis, largely because of variation in signal intensities between samples. FOXO targets were, however, also found in the AS list corroborating the complementarity of mechanisms affecting particular pathways in potentially mediating T cell suppression [34], [55]. In contrast to these previous studies, which measured stimulation dependent transcriptional alterations in Exon arrays of T cells (this did not include the inhibition approach we took), one study addressed differential accumulation of transcripts in T cells upon PI3K inhibition by a conventional micro-array analysis [29]. Some candidates identified in this study also appeared as RGs in ours (*IL-2B* and *CTLA-4* as up-, and IL-3 as down-regulated) while others – such as FoxP3 – did not appear in our lists. Most likely, substantial differences in cell material and stimulation protocols (they used naive CD4+ T cells and CD4+CD8− thymocytes as opposed to total CD3+ cells and harvested RNAs after a total stimulation period of 42 hrs rather than 24 hrs) may account to a major extent for discrepancies seen.

Availability and functions of splice regulatory factors are the most obvious targets in AS regulated downstream of PI3K activation (and ablation thereof). A few have been identified as PI3K effectors [20], [28] [21],[22],[62], yet their actual targets in regulating exonic inclusion/skipping mainly have not. It is thus unpredictable which PI3K effectors specifically account for AS from genes detected by the array and is there no algorithm allowing for a systematic search for exons affected (those shown in Figure 1 were manually determined based on published data). This is especially complicated by the fact that for many genes listed, exon usage and splice variants are not described, or if these are available, conflicting information is provided on enquiry of the NCBI or Affymetrix platforms. Thus, algorithms correcting for these inconsistencies and allowing for alignment of exonic inclusion/skipping as detected by the array need to be developed for optimal processing of information given. With regard to functional terms, it is, however, noteworthy that in addition to targeting genes directly involved in cell cycle progression (as also seen for RGs), AS appeared to preferentially affect genes involved in cytoskeletal dependent processes such as motility, invasion and chemotaxis, especially at the membrane proximal level (Figure S2).

On analysis of combined RG/AS candidates, it appeared that certain gene families and pathways were particularly subject to PI3K dependent regulation (Table 2), including the TNF/TNF-R and BCL superfamilies, or lipid metabolism, which has been referred to above. Genes involved in cancer progression were also readily identified (as outlined for *ATM* and *MXI* above), and these also included *WRAP53* as AS. Its gene product, WRAP53 (telomerase Cajal body protein, TCAB1) specifically interacts with *TERT* (AS on PI3K ablation) and essentially regulates telomerase trafficking, Cajal body formation and telomerase function and is thus of key importance for survival of particularly tumor cells [63],[64]. Interestingly, WRAP53 transcripts serve as natural anti-sense RNAs of p53 which regulate endogenous p53 mRNA and protein levels thereby supporting cellular apoptosis [38], [65]. Which of these WRAP53 functions applies to the regulation of T cell activation or survival is as yet unknown and needs detailed functional assessment. In addition to these proteins directly involved in cell division and survival, gene families regulating cytoskeletal dynamics also appear to be substantially affected (as exemplified for *VCL*, *CHN1* and *CALD1* above), which may be particularly important in regulating T cell functions in addition to those highlighted in Figure 4. The role of CD3γ within the TCR complex is virtually unknown, yet interestingly, this component as well as LCK has been found down-regulated in a microarray based study late after T cell activation [66]. Formal proof of the respective activity of especially the AS candidates in abrogating T cell responsiveness alone or in combination will have to be provided through recombinant expression and subsequent functional analysis as performed by us earlier for SIP110 [21].

## Supporting Information

Figure S1 **RNAs isolated from T cell enriched peripheral blood lymphocytes PMI/iono activated (S) or not (U) for the time intervals indicated were used as templates for RT-PCR detection of transcripts including exon 8 (lanes 2, 4, and 6).**
(EPS)

Figure S2 **Enriched functional categories in SI. Significantly enriched categories marked in red.** The analysis was performed using the Gene Set Analysis Toolkit V2 (http://bioinfo.vanderbilt.edu/webgestalt/).
(TIF)

Figure S3 **Enriched functional categories in down-regulated genes. Significantly enriched categories marked in red.** The analysis was performed using the Gene Set Analysis Toolkit V2 (http://bioinfo.vanderbilt.edu/webgestalt/).
(TIF)

Figure S4 **Enriched functional categories in up-regulated genes.** Significantly enriched categories marked in red. The analysis was performed using the Gene Set Analysis Toolkit V2 (http://bioinfo.vanderbilt.edu/webgestalt/).
(TIF)

Table S1 **Some of the genes from public databases showing alternative transcripts under normal versus T cell suppression conditions.**
(XLSX)

Table S2 **Primers used to amplify RG and AS transcripts (and variable (V) and constant (R) regions therein.**
(XLSX)

Table S3 **Genes found to be alternatively spliced.**
(XLSX)

Table S4 **Genes found to be regulated.**
(XLSX)

Table S5 **AS/RG genes involved in KEGG pathways chemokine signaling, T cell receptor signaling or leukocyte transendothelial migration.**
(DOCX)
