# Supplementary material for: Accumulation of Splice Variants and Transcripts in Response to PI3K Inhibition in T Cells
Source: PLoS One. 2013 Feb 1;8(2):e50695. doi: 10.1371/journal.pone.0050695 (PMC3562341; doi:10.1371/journal.pone.0050695)
Supplement: Table S5 — AS/RG genes involved in KEGG pathways chemokine signaling, T cell receptor signaling or leukocyte transendothelial migration. (DOCX) [file pone.0050695.s009.docx]

**Table S5**

| **Gene Symbol** | **logFC** |
| --- | --- |
| **regulated** |  |
| ADCY3 | -0.948 |
| CCL1 | -2.851 |
| CCR1 | -2.118 |
| CD3G | -1.129 |
| CD4 | -1.569 |
| CSF2 | -0.946 |
| CTLA4 | -1.466 |
| CX3CR1 | -2.2 |
| CXCL13 | -5.154 |
| CXCR3 | -1.215 |
| GNGT2 | -2.529 |
| ICOS | -0.882 |
| IL10 | -4.867 |
| ITGAL | -1.548 |
| MAPK13 | -1.302 |
| PPP3CA | 1.214 |
| CDKN1A | 0.983 |
| F11R | 1.280 |
|  |  |
| **spliced** |  |
| ADCY2 |  |
| ARRB1  CACNA2D2 |  |
| CLDN7 |  |
| CTNND1 |  |
| CXCR5 |  |
| CYBB |  |
| GNG5 |  |
| GRAP2 |  |
| ITGA4 |  |
| ITGAM |  |
| ITGB2 |  |
| LAP3 |  |
| LCK |  |
| MAPK3  NF1 |  |
| NOX3 |  |
| PLCB2 |  |
| PTPN6 |  |
| PTPRC |  |
| RAPGEF4 |  |
| SHC4 |  |
| TIAM1 |  |
| TIAM2 |  |
| VAV1  WHSC1 |  |
| VAV3 |  |
